# Quantitative dynamics of triacylglycerol accumulation in microalgae populations at single-cell resolution revealed by Raman microspectroscopy

**DOI:** 10.1186/1754-6834-7-58

**Published:** 2014-04-09

**Authors:** Tingting Wang, Yuetong Ji, Yun Wang, Jing Jia, Jing Li, Shi Huang, Danxiang Han, Qiang Hu, Wei E Huang, Jian Xu

**Affiliations:** 1 Single-Cell Center, CAS Key Laboratory of Biofuels and Shandong Key Laboratory of Energy Genetics, Qingdao Institute of BioEnergy and Bioprocess Technology, Chinese Academy of Sciences, 189 Songling Road, Qingdao, Shandong 266101, China; 2 Kroto Research Institute, University of Sheffield, Broad Lane, Sheffield, South Yorkshire S3 7HQ, United Kingdom; 3 Laboratory for Algae Research and Biotechnology, College of Technology and Innovation, Arizona State University, 7417 E. Unity Avenue, Mesa, Arizona 85212, USA

**Keywords:** Microalgae, Triacylglycerol, Single-cell Raman spectra, Bioprocess dynamics, Population heterogeneity

## Abstract

**Background:**

Rapid, real-time and label-free measurement of the cellular contents of biofuel molecules such as triacylglycerol (TAG) in populations at single-cell resolution are important for bioprocess control and understanding of the population heterogeneity. Raman microspectroscopy can directly detect the changes of metabolite profile in a cell and thus can potentially serve these purposes.

**Results:**

Single-cell Raman spectra (SCRS) of the unicellular oleaginous microalgae *Nannochloropsis oceanica* from the cultures under nitrogen depletion (TAG-producing condition) and nitrogen repletion (non-TAG-producing condition) were sampled at eight time points during the first 96 hours upon the onset of nitrogen depletion. Single *N. oceanica* cells were captured by a 532-nm laser and the SCRS were acquired by the same laser within one second per cell. Using chemometric methods, the SCRS were able to discriminate cells between nitrogen-replete and nitrogen-depleted conditions at as early as 6 hours with >93.3% accuracy, and among the eight time points under nitrogen depletion with >90.4% accuracy. Quantitative prediction of TAG content in single cells was achieved and validated via SCRS and liquid chromatography-mass spectrometry (LC-MS) analysis at population level. SCRS revealed the dynamics of heterogeneity in TAG production among cells in each isogenic population. A significant negative correlation between TAG content and lipid unsaturation degree in individual microalgae cells was observed.

**Conclusions:**

Our results show that SCRS can serve as a label-free and non-invasive proxy for quantitatively tracking and screening cellular TAG content in real-time at single-cell level. Phenotypic comparison of single cells via SCRS should also help investigating the mechanisms of functional heterogeneity within a cellular population.

## Background

Microalgae represent promising biomass feedstock for fuels because of their ability to grow rapidly and synthesize large amounts of storage chemical compounds from sunlight and carbon dioxide. They can be cultivated in non-arable land, non-potable water, and waste streams (such as flue gases and wastewaters), thus posing little competition to food crops while providing environmental benefits [1,2].

In response to environmental changes, microalgae efficiently modify lipid metabolism and result in a variety of cellular lipid patterns, including neutral lipids, polar lipids, wax esters, sterols and hydrocarbons [3]. Significant accumulation of neutral lipids in microalgae cells (for example 20 to 50% dry cell weight), mainly in the form of triacylglycerol (TAG), was demonstrated under unfavorable environmental or stress conditions such as nutrition limitation. TAGs serve primarily as carbon and energy storage in the form of dense lipid bodies in the cell and are considered as one ideal source for biodiesel [4]. Therefore, quantitative evaluation of the cell growth status and the cellular TAG content is essential for bioprocess monitoring and engineering for efficient and scalable biofuel production.

Sophisticated methodologies presently used for quantitation of cellular metabolite changes during bioprocesses, such as chromatography, mass spectrometry and nuclear magnetic resonance (NMR), are not only time and labor consuming, but also have mostly measured the stochastic average of the population, leaving phenotypic variations among individual cells masked [5-7]. However, functional diversity and phenotypic heterogeneity of microbial cellular behaviour have long been recognized among an isogenic population, such as those in cell growth, stress resistance, metabolites accumulation and other bioprocesses [8]. Such cell-to-cell variations of phenotypes have been shown to be crucial for the cells to adapt to fluctuating environments [9]. The averaged phenotypes in different populations may be similar, but their phenotypic distribution patterns at single-cell level can be dramatically different, which have significant impact on the populations’ functional stability and response to sudden changes such as stress or nutrient depletion [10,11]. Therefore, strategies for phenotypic measurement at single-cell resolution are of significant importance.

Raman microspectroscopy, which directly detects vibrations of biochemical bonds through the inelastic scattering by a laser light [12], provides a solution for rapid determination of metabolic fingerprint in real-time, as well as considerable improvements in speed [13]. Single-cell Raman microspectroscopy, combined with optical tweezers, enables the capture and subsequent acquisition of single-cell Raman spectra (SCRS) of individual live cells [14], thus serving as a biochemical fingerprint of a cell [15,16]. This label-free and *in situ* measurement property offers great advantages to the commonly used fluorescence based methods for the illustration of cellular lipid (such as Nile red) [17]. Related applications include confirmation of the existence of TAG in two algal species *Chlorella sorokiniana* and *Neochloris oleoabundans*[18], characterization of the structure and location of liquid hydrocarbons within *Botryococcus braunii* cells [19], calculation of total unsaturation and the number of double bonds in the hydrocarbon chains of microalgal lipids [20], as well as estimation of the total lipid abundance in *Chlorella vulgaris* pastes [21]. However, these previous studies have only focused on the general characteristics of cellular lipids and were not able to determine the cellular content of a particular lipid class of interest, either on single cells [18-20] or on pastes [21]. Moreover, these studies have required minutes for Raman signal acquisition in each SCRS measurement [18-20], which precluded many applications where throughput of measurement is important (such as temporal tracking of bioprocess). Quantitative assessment of specific lipid class (like TAG) at single-cell resolution with sufficient throughput is therefore yet to be achieved for the monitoring of bioprocess dynamics.

*Nannochloropsis* spp. are a group of unicellular oleaginous microalgae of particular industrial interests [22]. Here, using nitrogen-depletion triggered oil production of *Nannochloropsis oceanica* as a model, we sampled SCRS from nitrogen depletion (Group N-) and nitrogen repletion (Group N+) cultures at eight time points during the first 96 hours upon the onset of TAG accumulation. We show here that the SCRS, acquired within one second per cell, are able to discriminate cells between the two nutrition conditions at very early growth stage (6 h), and distinguish N-depleted cells among different time points with high accuracy. We further demonstrate quantitative prediction of TAG content in single cells via the SCRS, as well as reveal the dynamics of phenotypic heterogeneity, and the significant negative correlation between TAG content and lipid unsaturation degree among individual cells.

## Results and discussion

### Temporal tracking of triacylglycerol production in an isogenic population of microalgal cells

Group N- cells showed a slower growth than Group N + cells. The optical density at 750 nm (OD_750_) of N- Group cultures at 96 hours (the early stationary phase) reached 7.66 ± 0.05, which were approximately two-thirds of that of the Group N + cultures (OD_750_ = 12.09 ± 0.06). However, liquid chromatography-mass spectrometry (LC-MS) measurement showed that Group N- cells accumulated a significant amount of TAG, whereas little was observed in the Group N + cells. At 96 hours, the TAG content of Group N- cells reached 412.32 ± 13.13 mg g^-1^ dry weight while that of the Group N + cells remained low (1.73 ± 0.20 mg g^−1^ dry weight).

**Figure 1:**
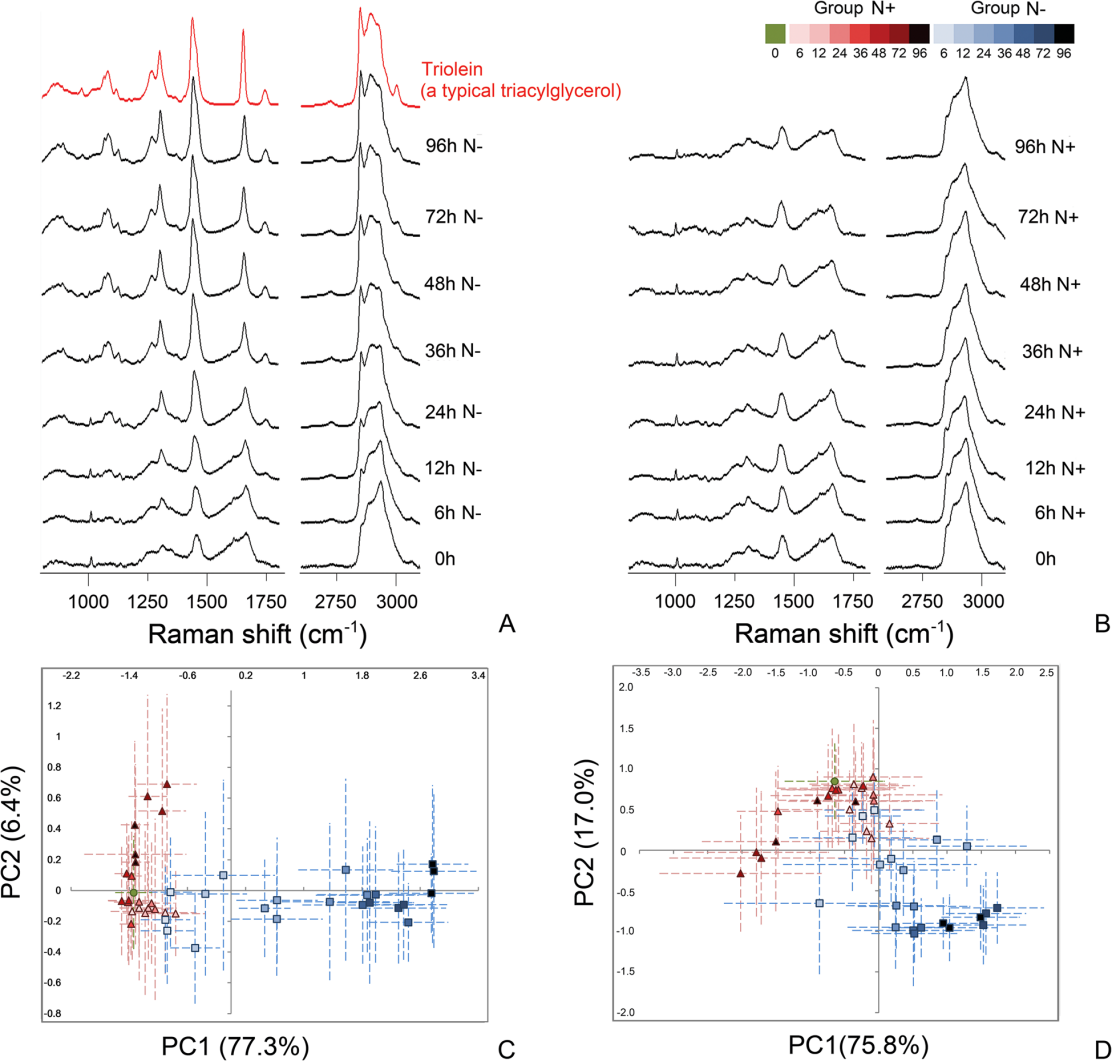
**Tracking the microalgal oil production via SCRS. (A)** Averaged SCRS of the 60 cells of Group N- at each time point as well as the Raman spectrum of triolein, a typical TAG species. **(B)** Averaged SCRS of the 60 cells of Group N + at each time point. **(C)** PCA scores plot derived from the fingerprint region. **(D)** PCA scores plot derived from the hydrocarbon region. Each symbol represents the average of twenty cells of a triplicate; the error bars represents SD of the twenty cells. Green diamond: cells at 0 h. Red triangle: cells of Group N+. Blue square: cells of Group N-. h: hours; PCA: principal component analysis; PC: principal component; SCRS: single-cell Raman spectra; SD: standard derivation; TAG, triacylglycerol.

For SCRS acquisition, a single *N. oceanica* IMET1 cell was optically trapped by a 532 nm laser and its Raman spectrum was recorded by the same laser. The typical acquisition time for a well−resolved spectrum of a microalgae cell was within one second. During the whole process, no loss of cell activity was observed, thus our measurement does not seem to have a significant negative impact on the health state of the cell (Additional file 1). This is also supported by our previous study which demonstrated that single bacterial cells were able to grow after trapping and SCRS measurement by a 532 nm laser [23]. In accordance with the difference in TAG production, both fingerprint region (800 to 1800 cm^−1^) and hydrocarbon region (2600 to 3100 cm^−1^) of SCRS exhibited very distinct patterns between Group N + and N− (Figure 1A and B). In the Group N− cells, intensities of all major lipid bands showed apparent increase along the cultivation time, including the bands representing chemical bonds attributed to chain unsaturation (1264 and 1656 cm^−1^) and saturation (1302, 1441, 2851 and 2889 cm^−1^) [20], and the 1746 cm^−1^ Raman band which indicates the ester specific chemical bond C=O stretching vibration specifically derived from TAG [24]. Meanwhile, intensities of protein−relative bands (for example 1005, 1200–1350, and 1600–1700 cm^−1^) [25] decreased in the N- group cells (Table 1). Note that all main peaks in the SCRS profiles of Group N- cells at the later time points starting from 36 hours were consistent with the profiles of triolein, a typical TAG species (Figure 1A). In contrast, the Group N + cells showed no change with these bands during the whole bioprocess (Figure 1B). These findings suggested a continuous TAG production by the cells, accompanied with a deficiency of protein biosynthesis under a nitrogen-deficiency condition, which was consistent with previous studies [26,27].

**Table 1:** List of major Raman bands discriminating between different states of the cells

**Raman bands (cm**^**−1**^**)**	**Trend with time in Group N- cells**	**Biological assignment/interpretation**
**1066**	↑	Lipid, Alkyl C—C gauche stretches
**1080**	↑	Carbohydrate, Carbohydrate C—O—H bending
**1125**	↑	Carbohydrate, C—O—H deformation, C—O and C—C stretches
**1264**	↑	Lipid, Alkyl =C—H cis stretches
**1302**	↑	Lipid, Alkyl C—H_2_ twist
**1441**	↑	Lipid, Alkyl C—H_2_ bend
**1656**	↑	Lipid, Alkyl C=C stretches
**1746**	↑	Lipid, Ester C=O stretches
**2851**	↑	Lipid, carbohydrate, C—H_2_, C—H_3_ asymmetric and symmetric stretches
**2889**	↑	
**1003**	↓	Protein, Phenylalanine ring breath
**1610**	↓	Protein Amide I

Up and down arrows indicate the Raman bands which increased or decreased in intensity during the growth of Group N- cells respectively.

**Figure 2:**
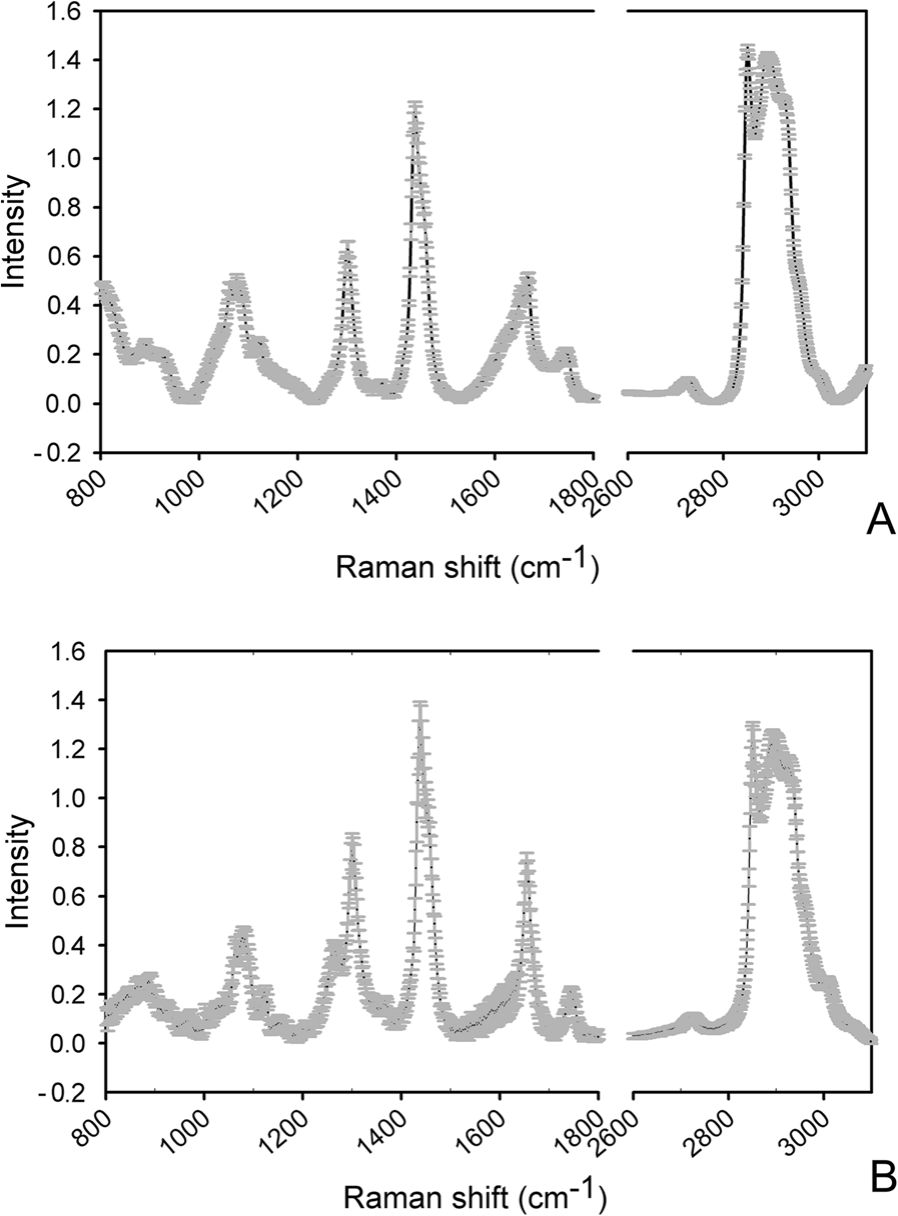
**Comparison of variation of SCRS. (A)** Variation of Raman spectra of one Group N- cell at 48 hours for 20 continuous measurements. **(B)** Variation of Raman spectra of 20 Group N- cells at 48 hours as an example. SD is shown in gray. SCRS: single-cell Raman spectra; SD: standard derivation.

The variation of Raman spectra between individual cells were observed within a population. To verify the reproducibility of the method, Raman profiles were generated by 20 continuous measurements on one cell of Group N- at 48 hours (Figure 2A), and compared to the Raman profiles of each of the 20 cells of all Group N- triplicates at the eight time points (Figure 2B). Variation was quantified using a standard deviation of the mean (SDM) as described previously [28]. The SDM values of the fingerprint region and hydrocarbon region for 20 measurements of one cell were 0.070 and 0.014 respectively, while those for 20 cells at eight time points were 0.254 ± 0.070 and 0.101 ± 0.033 respectively, showing that the variation of 20 measurements was much smaller than those of 20 cells. These results demonstrate the high reproducibility of SCRS measurement and reliable distinction of the biochemical property between the cells.

Principal component analysis (PCA) was a commonly used approach for the evaluation of the ordination of different observations. It is shown by PCA scores plots, based either on the fingerprint region or the hydrocarbon region, that while Group N + cells aggregated, Group N- cells showed a clear differentiation according to their growth time. The first principal component (PC1) explained 77.3% and 75.8% of the total variance respectively (Figure 1C and D). The trend of differentiation was in accordance with the process of TAG accumulation, indicating that it may be responsible for the distinguishing of cells at different growth stages.

SCRS provides a sensitive biochemical ‘fingerprint’ to measure cell-to-cell variability. In this study, we have applied an optimized Raman microscope to obtain each SCRS within one second. The improved Raman system was equipped with a short Raman light path, low noise and sensitive electron multiplying charge coupled device (EMCCD) for the Raman signal detection, and an appropriate incident laser of 532 nm [29]. Its sensitivity and throughput was over two orders of magnitude higher than a recent study whereby 120 seconds of prerequisite photo-bleaching and 10 seconds of the Raman signal acquisition were required for each SCRS measurement [20], and thus allows for screening of a large amount of cells in a short time, which is critical for the continuous monitoring of the entire bioprocess.

### Discrimination of cells from different temporal phases during the oleaginousness process

The significant change of Raman spectra of individual cells enabled these cells to be further compared and discriminated. Linear discriminant analysis (LDA) in combination with PCA achieved highly accurate classification between cells under N- or N + condition at each time point. For the analysis using the fingerprint region of the Raman spectra, the misclassification rate was 6.7% at 6 h and decreased to 3.3% at 12 h and 24 h (validation data). For cells at each time point since 36 h, all cells were classified to N- or N + groups correctly. The spectra of the hydrocarbon region also generated 100% correct classification at each time point starting from 24 h, although the misclassification rates at the earlier time points were relatively higher (16.7% at 6 h and 6.7% at 12 h, validation data) (Table 2).

**Table 2:** Predictive modeling of nutrition condition of single cells

**Fingerprint region**							
	6 h	12 h	24 h	36 h	48 h	72 h	96 h*
**Number of PCs used in LDA**	9	9	2	2	2	2	2
**Variations explained by these PCs (%)**	67.5	82.0	72.6	89.1	91.1	85.7	93.6
**MCR of calibration data (%)**	2.2	2.2	1.1	1.1	1.1	1.1	0
**MCR of validation data (%)**	6.7	3.3	3.3	0	0	0	0
**Hydrocarbon region**							
	6 h	12 h	24 h	36 h	48 h	72 h	96 h*
**Number of PCs used in LDA**	10	12	5	2	2	2	2
**Variations explained by these PCs (%)**	89.1	95.8	94.9	96.3	94.7	97.9	97.2
**MCR of calibration data (%)**	2.2	0	0	0	0	0	0
**MCR of validation data (%)**	16.7	6.7	0	0	0	0	0

*For both calculations, one outlier was excluded from the training set at 96 h.

PC-LDA results were shown for discriminating cells between Groups N- and N + at each time point. h: hours; LDA: linear discriminant analysis; MCR: misclassification rate by leave-one-out cross validation (LOOCV); PC: principal component.

Principal component-linear discrimination analysis (PC-LDA) was also performed to temporally discriminate all Group N- cells between the seven different culture stages (6, 12, 24, 36, 48, 72 and 96 hours) under nitrogen depletion and the cells at starting time point (0 hours). The first 20 principal components (PCs), which accounted for more than 90% of the whole variance, were used in the analyses. Using the fingerprint region of the Raman spectra, low misclassification rates of 6.1% for calibration data and 7.5% for validation data were achieved. In total, 31 out of 480 cells were classified incorrectly (assigned to a different growth stage from the actual one). PC-LDA using the hydrocarbon region of the Raman spectra generated similar results, with a misclassification rate of 10.0% for calibration data and 7.5% for validation data (Table 3).

**Table 3:** Predictive modeling of growth stage of single cells

	**Fingerprint region**		**Hydrocarbon region**	
	**Calibration data**	**Validation data**	**Calibration data**	**Validation data**
	**Number of cells misclassified (MCR)**		**Number of cells misclassified (MCR)**	
**0 h**	0 (0)	0 (0)	0 (0)	1 (6.7%)
**6 h**	0 (0)	1 (6.7%)	4 (8.9%)	2 (13.3%)
**12 h**	3 (6.7%)	1 (6.7%)	5 (11.1%)	1 (6.7%)
**24 h**	4 (8.9%)	1 (6.7%)	6 (13.3%)	1 (6.7%)
**36 h**	3 (6.7%)	2 (13.3%)	6 (13.3%)	1 (6.7%)
**48 h**	6 (13.3%)	1 (6.7%)	5 (11.1%)	0 (0)
**72 h**	1 (2.2%)	2 (13.3%)	6(13.3%)	2 (13.3%)
**96 h**	5 (11.1%)	1 (6.7%)	4 (8.9%)	1 (6.7%)
**Total**	22 (6.1%)	9 (7.5%)	36 (10.0%)	9 (7.5%)

PC-LDA results were shown for discriminating Group N- cells at different time points. h: hours; MCR: misclassification rate by leave-one-out cross validation (LOOCV).

Dynamic monitoring of the nutrient status of microalgae cells is important for manipulation of nutrient availability in algal biofuel production in order to optimize yields [30]. Single-cell Raman microspectroscopy was previously employed to distinguish the eukaryotic chlorophyte alga *Dunaliella tertiolecta* cells from N-replete and N-starved conditions after four days adaptation based on chlorophyll a and beta-carotene bands [31]. Here, we showed that SCRS is able to distinguish between N-depleted and N-replete conditions in oleaginous microalgae cells from as early as six hours under nitrogen depletion, which showed a significant improvement in sensitivity. Furthermore, we demonstrated for the first time the ability of SCRS to temporally discriminate among the N-depleted cells (6, 12, 24, 36, 48, 72 and 96 hour cultivations), which is important for the continuous monitoring of the oleaginousness process.

### Quantitative dynamics of triacylglycerol content in single cells

To further investigate the TAG accumulation in the cells, we have developed a partial least square regression (PLSR) model to quantify the TAG content in individual Group N- cells at different time points. PLSR is a powerful tool in the development of calibration models for determination of particular parameters, with the advantage of dealing with spectra-containing overlapping signals and noises [32]. It is also widely used in the quantification of specific components in complicated chemical products [33] or fermentation broth [34]. Spectra of the fingerprint region were selected for modeling due to their better performance in PCA and LDA as described above.

**Figure 3:**
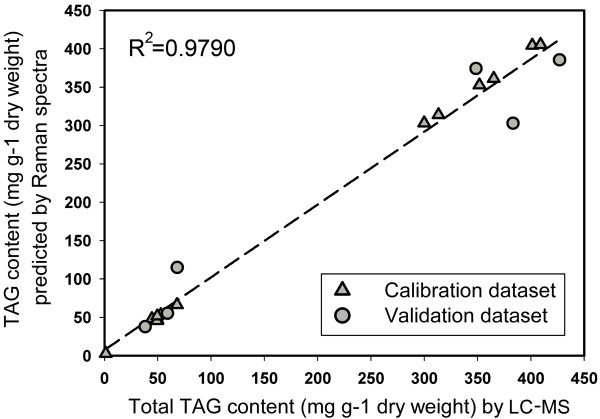
**Establishment and validation of the PLSR model for TAG content prediction.** The predicted TAG content of each population by PLSR model was plotted versus the TAG content of the corresponding culture measured by LC-MS methods. LC-MS: liquid chromatography-mass spectrometry; PLSR: partial least square regression; TAG: triacylglycerol.

**Figure 4:**
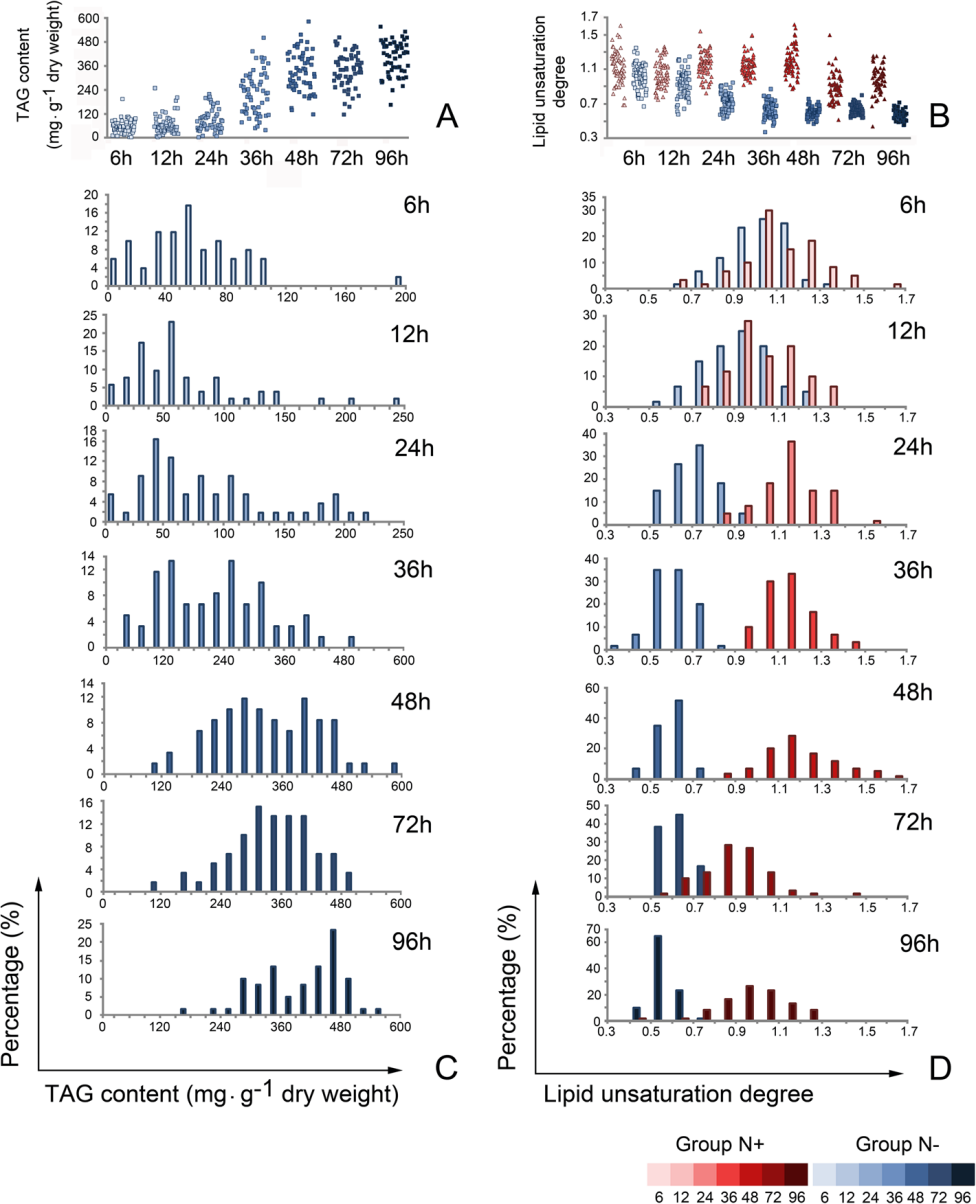
**Quantitative analysis of TAG content and lipid unsaturation degree in single cells. (A)** TAG content of individual cells as predicted by PLSR. Each square represents one Group N- cell. **(B)** Lipid unsaturation degree as determined by I_1656_/I_1441_. Each triangle represents one Group N + cell, and each square represents one Group N- cell. **(C)** Distribution of TAG content of Group N- cells at each time point. X axis is the predicted TAG content (mg g^−1^ dry weight), Y axis is the percentage of cells. Since TAG content is increasing sharply, the range of X axis is different between time points. **(D)** Distribution of lipid unsaturation degree of Group N- or Group N + cells at each time point. X axis is the lipid unsaturation degree, Y axis is the percentage of cells. h: hours; PLSR: partial least square regression; TAG: triacylglycerol.

For the 0 hour and Group N- cultures, the total TAG content determined by LC-MS increased from 1.23 ± 0.27 mg g^−1^ dry weight at 0 hours to 412.32 ± 13.13 mg g^−1^ dry weight at 96 hours, a 335-fold increase in TAG. The PLSR model was established and validated using the averaged SCRS of all cells at 0 hours, and averaged SCRS of each triplicate at 6, 12, 24, 48, 72 and 96 hours, as well as the TAG content of the corresponding cultures by LC-MS. The optimal number of partial least square (PLS) components was set as seven. A highly reliable regression model was generated, with a mean squared error of calibration (MSEC) of 0.0854, with the correlation coefficient value (R^2^) of 0.9997 for calibration dataset and 0.9465 for validation dataset. The overall correlation coefficient value (R^2^) reached 0.9790 (Figure 3). The TAG content of each Group N- cell at 6, 12, 24, 36, 48, 72 and 96 hours was then estimated through their Raman spectra by the established model. Among all 420 cells at the seven time points, the predicted TAG content of 22 cells were lower than the measured TAG content value of the 0 hour culture (1.23 mg g^−1^ dry weight), including nine cells at 6 hours, eight cells at 12 hours and five cells at 24 hours. These cells were thus regarded as lower than the detection threshold and were discarded in the following analysis. The TAG content of individual cells also showed a continuous increase during cell growth (Figure 4A). The mean value of predicted cellular TAG contents of each triplicate showed significant correlation with the TAG content of the corresponding cultures by LC-MS (Pearson correlation, *r* = 0.9864, *P* < 0.01), suggesting that while revealing varied TAG content of individual cells, SCRS could also be an ideal representative for the overall feature of the corresponding population.

Lipid unsaturation degree is also a key parameter in the evaluation of biofuels. At the single-cell level, the changes of lipid unsaturation degree accompanying the TAG accumulation can be measured by I_1656_/I_1441_ of the Raman spectra, as reported previously [20]. To validate this method, the mean value of lipid unsaturation degree by SCRS of each of the 20 cells was calculated and compared with the population level lipid unsaturation degree calculated by LC-MS (Additional file 2). A significant positive correlation was revealed (Pearson correlation, *r* = 0.906, *P* < 0.01), supporting the ability of I_1656_/I_1441_ to estimate the unsaturation degree of total cellular lipid in the cell. Based on this method, we found that for individual Group N- cells, the degree of lipid unsaturation decreased sharply before 36 hours and stayed stable later on, showing a significant change during the growth (analysis of variance (ANOVA) test, *P* < 0.01). However for individual Group N + cells, lipid unsaturation was relatively constant during 0 to 48 and 72 to 96 hours respectively, but showed an obvious decrease between 48 and 72 hours. The degree of lipid unsaturation of Group N- cells was significantly lower than Group N + cells at each time point after 6 hours (Student t test, *P* < 0.01) (Figure 4B).

The estimated TAG content of each cell in Group N- showed a significant negative correlation with its lipid unsaturation degree (Spearman’s rank correlation coefficient *p* = **−**0.800, *P* < 0.01; Additional file 3: Figure S1A), which was consistent with the observation at the population level (TAG content of each Group N- culture versus its lipid unsaturation degree determined by LC-MS, Spearman’s rank correlation coefficient *p* = **−**0.977, *P* < 0.01, Additional file 3: Figure S1B). Thus individual cells with a higher TAG content tend to possess more saturated lipids. Since both TAG contents and lipid unsaturation degree are key parameters in the evaluation of biofuels, the SCRS measurements can be used to screen for biofuel-producing feedstock or processes.

**Figure 5:**
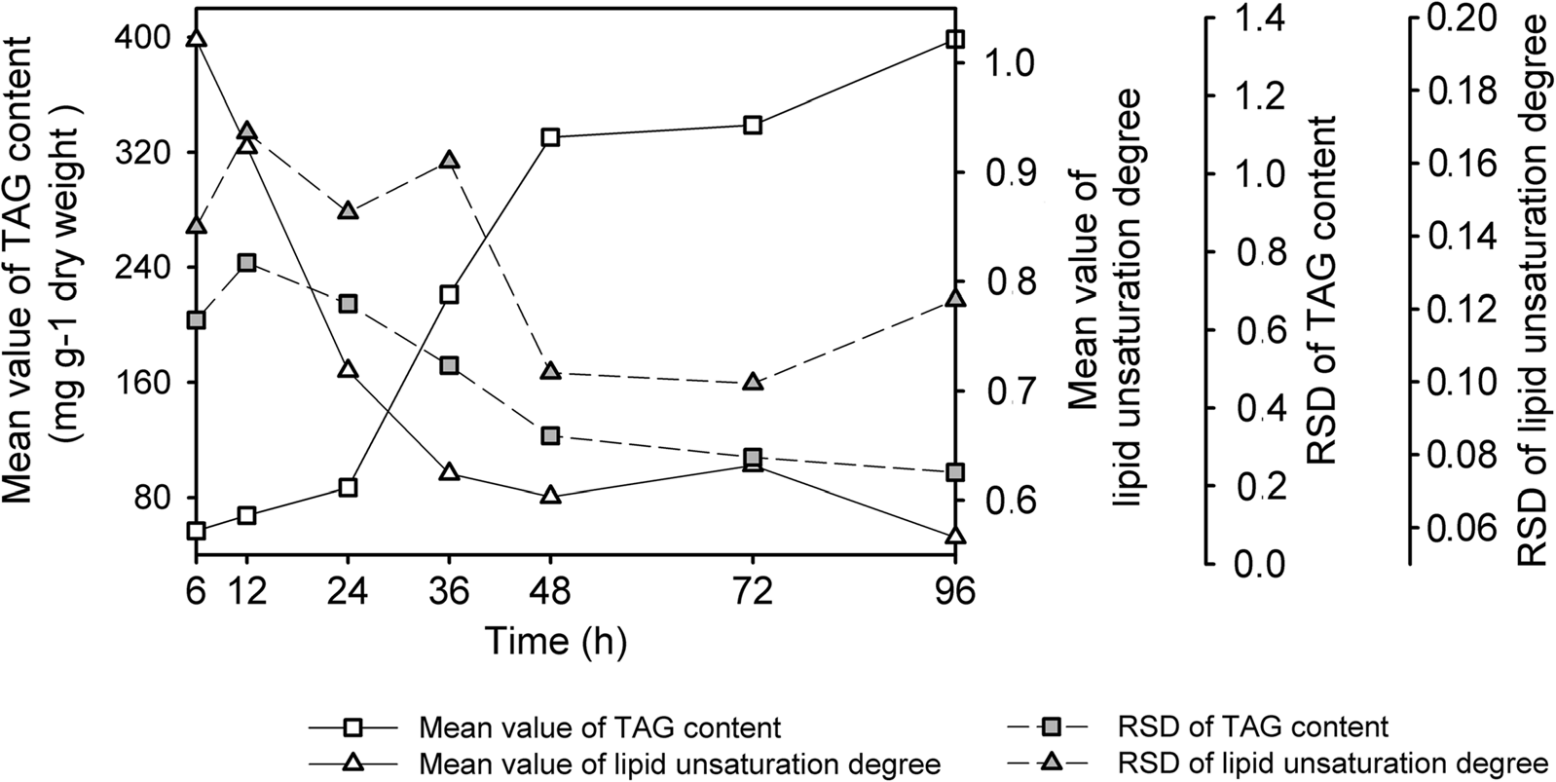
**Comparison of TAG content and lipid unsaturation among Group N- cells.** Mean value and RSD value of predicted TAG content and lipid unsaturation degree of Group N- cells at each time point were compared to show temporal patterns of population heterogeneity. h: hours; RSD: relative standard deviation; TAG: triacylglycerol.

Our findings further enabled modeling of the dynamics of population heterogeneity in terms of particular traits of interest. At each time point, both lipid unsaturation degree and TAG content of individual cells showed an approximate normal distribution, indicating the internal heterogeneity on the population level (Figure 4C and D). We have used the relative standard deviation (RSD) of measurements of multiple cells at the same time point to represent the population heterogeneity. During the continuous increase of TAG contents in Group N- cells over time, the heterogeneity of TAG content decreased accordingly. On the other hand, while the heterogeneity of lipid unsaturation in a population exhibited no monotonic changes in Group N + up to 96 hours, that of Group N- decreased continuously after 48 hours (Table 4). Both suggested that nitrogen-depletion stress might be a strategy for the homogenization of certain cell components in an algal population (Figure 5).

**Table 4:** Heterogeneity of TAG and lipid unsaturation degree as represented by RSD

**RSD value**	**6 h**	**12 h**	**24 h**	**36 h**	**48 h**	**72 h**	**96 h**
Predicted TAG content (Group N-)	0.602	0.748	0.643	0.485	0.305	0.249	0.211
Lipid unsaturation degree (Group N-)	0.140	0.166	0.144	0.158	0.100	0.097	0.102
Lipid unsaturation degree (Group N+)	0.168	0.150	0.122	0.104	0.141	0.182	0.162

RSD: relative standard deviation; TAG: triacylglycerol; h: hours.

Recently, researchers have used Raman spectra to non-specifically quantify the total cellular lipids in microalgae cell pastes (*Chlorella* sp.) using signal intensities of the range 2845–3107 cm^−1^[21]. Here, we have for the first time demonstrated the quantification of a particular lipid component of biotechnology interest in individual cells. In both industry and laboratorial cultures, synchronous growth is frequently desirable for optimum yield coefficients and yet can be difficult to sustain [8]. Moreover, the degree of metabolic and phenotypic heterogeneity can be different among cellular populations as it depends on species-specific genetic traits, and physiological features under particular microenvironment and culture conditions [35]. The ability of our method to track the degree of heterogeneity in the TAG level at single-cell resolution, when coupled with techniques such as phased cultures, tailor made biofilms and microfluidics devices [35,36], can be used to identify the most efficient oil producers, separate them from others and adapt them to favored conditions. It should therefore be of value to bioprocess optimization and control, as well as to the screening and engineering of oleaginous microalgae strains for enhanced oil productivity.

Moreover, recent studies have suggested that the cellular heterogeneity has profound biological implications [37]. Tracking of phenotypic traits and gene expression patterns at single-cell resolution can provide a deeper view of the population heterogeneity and help to understand the complicated behaviors of the populations and consortia [10]. Several studies have coupled gene expression and physiological parameters in individual cells. For example, analyses of volume, cell cycle and gene expression of individual yeast cells led to the identification of two mechanisms that regulate cell-to-cell variation in pathway capacity [38]. However, the connection between the stochastic gene expression and the physiological diversity within a population remains elusive [9]. Here, using the oleaginous microalga *N. oceanica* as a model, we have demonstrated rapid, label-free quantification of a particular lipid component of biotechnological interest in individual cells. For a given microalgae cell, the TAG concentration can then be coupled with gene expression analysis via Raman-activated cell sorting [29] and subsequent quantitative PCR or mRNA sequencing methods [39]. The phenotypes and genotypes at the level of single cells can thus be tested for correlations with each other and then compared to those at the population level [40], so as to elucidate the molecular mechanism underlying the phenotypic heterogeneity in an oleaginous cellular population.

Since SCRS offers real-time monitoring and bioprocess diagnosis capabilities without prior knowledge of any cellular component or metabolite as biomarker, and needs no labeling to the cell, its application may not be limited to the investigation of TAG accumulation, but also to bio-prospecting of novel phenotypes in yet-to-be-culture or mutant cells. Further development in hardware and software, such as microfluidics devices, and more statistical tools should allow for improvement of the specificity, sensitivity, spatial resolution and throughput of SCRS, and establish it as a general approach for characterization, screening, isolation and in-depth analysis of microbial cells or live-cell-mediated processes for broad applications [11,41].

## Conclusions

In this study, we demonstrated that SCRS for one individual microalgal cell acquired at sub-second level by 532 nm Raman spectroscopy are able to provide sufficient phenotypic information for the quantitative evaluation of its TAG content and the lipid unsaturation degree. In addition, comparison of SCRS among cells revealed the phenotypic heterogeneity of cells within an isogenic population. Therefore, SCRS is able to serve as a proxy for rapidly, quantitatively, and non-invasively tracking and screening the dynamics of cellular TAG contents in real-time, at single-cell level. It should also help with the investigation of the mechanisms behind functional heterogeneity within a cellular population.

## Methods

### Microalgal growth

*N. oceanica* IMET-1 cells were inoculated into a modified f/2 liquid medium with 4 mM NO_3_^−^ under continuous light (approximately 50 μmol photons m^−2^ s^−1^) at 25°C and aerated by bubbling with a mixture of 1.5% CO_2_ in air. Composition of the medium was as below: 200 g/L KNO_3_, 25 g/L NaH_2_PO_4_ · 2H_2_O, 5 g/L FeCl_3_ · 6H_2_O, 4.5 g/L EDTA and 1 mL of trace element solution (pH = 7.6). The trace element solution was comprised of 0.4 mg/L MnCl_2_ · 4H_2_O, 0.02 mg/L Na_2_MoO_4_ · 2H_2_O, 0.02 mg/L CoCl_2_ · 6H_2_O, 0.02 mg/L CuSO_4_ · 5H_2_O, 0.04 mg/L ZnSO_4_ · 7H_2_O, 1 μg/L vitamin B_12_, 1 μg/L biotin and 200 μg/L thiamine · HCl. Cell density (OD_750_) was determined in triplicate on a UV/Vis spectrophotometer (Beijing Purkinje General Instrument Co., Ltd., Beijing, China). Early-logarithmic phase algal cells were collected, washed three times with axenic seawater and re-inoculated with equal concentration in annular glass columns, either under the same condition as above (nitrogen-replete, or Group N+) or with no NO_3_^-^ supplemented (nitrogen-depleted, or Group N-). Re-inoculation was performed in triplicate.

### Single-cell Raman microspectroscopy

Cell aliquots were collected right before re-inoculation (at 0 hours), and from each triplicate of Group N + and Group N- at seven time points afterward: 6, 12, 24, 36, 48, 72 and 96 hours. Each cell sample was washed and re-suspended with ddH_2_O to avoid the high noise introduced by the culture media, and was immediately loaded into a capillary tube (50 mm length × 1 mm width × 0.1 mm height, Camlab, Cambridge, UK) for measurement [42]. The Raman spectra of individual cells were acquired using a Raman Activated Cell Sorting system (RACS, Wellsens Inc, Beijing, China), which was equipped with a confocal microscope with a 50 × PL magnifying dry objective (NA = 0.55, BX41, Olympus UK Ltd., Southall, UK) and a 532 nm Nd:YAG laser (Ventus, Laser Quantum Ltd, Stockport, UK). The laser power out of the objective was 50 mW. Individual microalgae in the capillary tube was captured and measured by the same 532-nm laser. The whole process, including a single-cell capture and Raman measurement, was performed within one second. The scattered photons were collected by a Newton EMCCD (Andor, Belfast, UK) utilizing a 1600 × 200 array of 16 μm pixels with thermoelectric cooling down to −70°C for negligible dark current. Each Raman spectrum was acquired between the range 3256 cm^−1^ and 273 cm^−1^, with a spectral resolution of 2 cm^−1^ achieved by a 300 groove mm^−1^ grating in the spectrograph. Sixty individual cells were measured for the sample at 0 hours, and twenty were measured in each of all other samples. For each sample, a background spectrum was generated as the average of five spectra acquired from the liquid around the cell.

Pre-processing of raw spectra was performed with LabSpec 5 (HORIBA Scientific, Orsay, France), including background subtraction and the baseline correction by a polynomial algorithm with a degree of seven. Two regions of the spectra: the biochemical fingerprint region (800 cm^−1^ to 1800 cm^−1^) and the hydrocarbon region (2600 cm^−1^ to 3100 cm^−1^), were extracted for further analyses in order to extract useful information contained in Raman bands from the useless noise [28,43]. For both regions a spectrum was normalized via division by its area. The lipid unsaturation degree in each cell was calculated as the ratio of Raman intensity of the C=C stretch and the intensity of CH_2_ bend, namely I_1656_/I_1441_, as described previously [20].

### Determination of cellular lipid content by LC-MS

LC-MS was performed on the same cell aliquots which were sampled for SCRS, at 0 hours and each triplicate of the six time points: 6, 12, 24, 48, 72 and 96 hours. A quantitative LC-MS method was used to determine the cellular content of molecular lipid species belonging to nine major glycerolipid classes including TAG, monogalactosyldiacylglycerol (MGDG), digalactosyldiacylglycerol (DGDG), sulfoquinovosyldiacylglycerol (SQDG), diacylglycerol-O-(N,N,N–trimethyl)-homoserine (DGTS), phosphatidylcholine (PtdCho), phosphatidylethanolamine (PE), phosphatidylglycerol (PG) and phosphatidylinositol (PI). These nine main lipid classes, including 74 lipid species, constitute the majority of total lipids in *Nannochloropsis* cells [44,45]. Total lipids were extracted with chloroform:methanol (2:1, w/w) and recovered in chloroform: methanol (1:1) before being loaded for quantification. An Agilent 6460 triple quadruple electrospray ionization mass spectrometer equipped with a 1260 high performance liquid chromatography (Agilent Technologies, Santa Clara, CA, United States) was used for LC-MS analysis. PtdCho, PE, DGTS, TAG, MGDG and DGDG were detected at the positive mode, with the mobile phases of methanol : acetonitrile: H_2_O (19:19:2; A) and isopropanol (B) containing 0.1% formic acid and 10 mM ammonium acetate. PI, PG and SQDG were detected at the negative mode with the mobile phases of 85% methanol (A) and isopropanol containing 0.025% NH_4_OH. The LC gradients for positive mode were as follows: 0 minutes, 90% A and 10% B; 5 minutes 90% A and 10% B; 25 minutes, 60% A and 40% B; 60 minutes 45% A and 55% B; 66 minutes, 45% A and 55% B; 68 minutes 90% A and 10% B. For negative mode, the LC gradients were as follows: 0 minutes, 95% A and 5% B; 15 minutes, 85% A and 15% B, 22 minutes 45% A and 55% B; 42 minutes, 45% A and 55% B; 44 minutes 95% A and 5% B. The flow rate was 0.2 mL min^−1^. Nitrogen was used as nebulizing gas (at 0.3 Bar) and a dry gas (4 L min^−1^ at 200°C). The spray capillary voltage was 3700 V for the negative ion mode and 4200 V for the positive ion mode. For quantification of lipid content, TAG 51:0 (17:0/17:0/17:0), MGDG 36:0 (18:0/18:0), DGDG 36:0 (18:0/18:0), PE 31:1 (14:1/17:0) and PG 37:4 (17:0/20:4) were used as the internal standard (ITSD) for TAG, MGDG, DGDG, PE and PG respectively. PtdCho 37:4 (17:0/20:4) was used as ITSD for both PtdCho and DGTS, and PI 37:4 (17:0/20:4) for both PI and SQDG. TAG 48:3 (16:1/16:1/16:1), TAG 50:1 (16:0/18:1/16:0), TAG 52:2 (18:1/16:0/18:1) and TAG 54:3 (18:1/18:1/18:1) were used as calibration standards for 48 carbon, 50 carbon, 52 carbon and 54 carbon TAG quantifications, respectively. MGDG 34:6 (16:3/18:3), DGDG 36:3 (18:3/18:3), PE 36:1 (18:0/18:1), PG 36:1 (18:0/18:1), PtdCho 36:2 (18:1/18:1) and DGTS 32:0 (16:0/16:0) were used as calibration standards for their corresponding lipid class.

The ratio of the number of C=C bonds and the number of CH_2_ bonds of each lipid species was calculated to represent the unsaturation degree of this lipid species as previously described [20]. The unsaturation degree of the total cellular lipid of each culture was then calculated by multiplying the unsaturation degree of each lipid species by its relative abundance in the cellular extract (μmol/g dry weight).

### Chemometrics analyses

The normalized fingerprint and hydrocarbon regions of spectra from each cell were used separately for PCA based on Euclidean distances [46], followed by LDA based on the principal components extracted by PCA. Both PCA and LDA use linear combinations of the original variables (wavenumbers of the spectra) as PCs to characterize the ordination of samples or to discriminate two or more classes of samples. The first few PCs were used for LDA instead of the original variables in order to reduce the dimensionality of the variation. For the LDA-discriminating cells between N + and N- conditions at separate time points, 15 out of 20 cells from each triplicate of the two conditions were randomly selected and combined for the construction of a training dataset (n = 90), and the rest of the cells were used to form a test dataset (n = 30). For the LDA-discriminating cells at 0 hours and Group N- cells at different time points, 45 out of 60 cells at 0 hours and 15 out of 20 cells from each triplicate at the seven time points afterwards, were randomly selected and combined for the construction of a training dataset (n = 360), and the rest of the cells were used to form a test dataset (n = 120). Leave-one-out cross validation (LOOCV) was used to evaluate the reliability of the LDA model based on the training dataset, followed by challenging the model with the test dataset. The misclassification rates of both the training and test dataset were calculated to determine an optimal number of PCs [47]. The test dataset was rotated into a new dataset of PCs by the loadings of the PCA of the training dataset as described previously, so as to convert two datasets in the same spectral space [28].

SCRS data is used to construct a PLSR model together with the TAG content of corresponding samples determined by LC-MS. By relating two datasets X (SCRS in our study) and y (TAG content by LC-MS in our study) by means of regression, PLSR performs a multivariate calibration in order to establish a linear model which enables the prediction of y from measured dataset X. In the regression process, decomposition of X is performed under the consideration of y in a simultaneous analysis of the two datasets [48]. Specifically, the SCRS of all 60 cells at 0 hours, and that of each of the 20 cells from triplicates at 6, 12, 24, 48, 72, and 96 hours were averaged separately, generating data of 19 combined Raman spectra as a matrix (designated as X). Among them, two of the triplicates at 6, 12, 24, 48, 72 and 96 hours were randomly selected and combined with the 0 hour data to form a training dataset for the calibration of the model (Xc, n = 13), and the rest were used as a test dataset for validation (Xv, n = 6). Correspondingly, TAG content of 0 hours culture and each triplicate culture at 6, 12, 24, 48, 72, and 96 hours were measured by LC-MS, generating data of another 19 values as a vector (designated as y), also including the training dataset (yc, n = 13) and the test dataset (yv, n = 6). Firstly, the PLSR model was established using Xc and yc data. Secondly, Xv data and the function of the established model were used to predict the yv value. The predicted yv value was compared with the measured yv value. Reliability of the model was assessed by the MSEC of the training set by LOOCV, as well as the squared correlation coefficient (R^2^) between predicted and measured y values in both the training set and the test set. Finally, the function was utilized for the prediction of TAG content in single Group N- cells (as y value) at each time point between 6 and 96 hours using individual SCRS (as X data). PCA, LDA and PLSR were performed with Matlab R2010a (Mathworks, Natick, Massachusetts, United States). Other statistical analyses were performed with SPSS Statistics 17.0 (SPSS Inc, Chicago, Illinois, United States).

## Abbreviations

DGDG: digalactosyldiacylglycerol; DGTS: diacylglycerol-O-(N,N,N–trimethyl)-homoserine; EMCCD: electron multiplying charge coupled device; ITSD: internal standard; LC-MS: liquid chromatography/mass spectrometry; LDA: linear discriminant analysis; LOOCV: leave-one-out cross validation; MCR: misclassification rate; MGDG: monogalactosyldiacylglycerol; MSEC: mean squared error of calibration; NMR: nuclear magnetic resonance; OD: optical density; PC: principal component; PCA: principal component analysis; PE: phosphatidylethanolamine; PG: phosphatidylglycerol; PI: phosphatidylinositol; PLSR: partial least square regression; PtdCho: phosphatidylcholine; RSD: relative standard deviation; SCRS: single-cell Raman spectra; SD: standard deviation; SDM: standard deviation of the mean; SQDG: sulfoquinovosyldiacylglycerol; TAG: triacylglycerol.

## Competing interests

The authors declare that they have no competing interests.

## Authors’ contributions

TW, YJ, WH and JX designed the research. TW and SH analyzed data. YJ and YW performed Raman spectrometry experiments. JJ, JL, DH and QH performed algal cultivation and LC-MS experiments. TW, WH and JX wrote the manuscript. YJ, YW, JJ, JL, SH, DH and QH revised the manuscript and provided critical suggestions. All authors read and approved the final version of the manuscript.

## Supplementary Material

Additional file 1 **Impact of the laser power on microalgal cell.** A single *N. oceanica* IMET1 cell was optically trapped by a 532 nm laser and its Raman spectrum was recorded in one second by the same laser. No loss of cell activity was observed, our measurement thus does not seem to have a significant negative impact on the health state of the cell. Click here for file

Additional file 2 **Cellular content of the 74 lipid species in cultures under nitrogen depletion conditions.** The cellular contents of each species of the nine lipid classes (TAG, DGDG, MGDG, DGTS, SQDG, PI, PG, PC and PE) along the time course were listed. Click here for file

Additional file 3 **Correlation between the TAG content and lipid unsaturation degree at single-cell level and population level. (A)** Correlation between TAG content and lipid unsaturation degree at the single-cell level as measured by SCRS. Each dot represents one cell in the N- cultures. **(B)** Correlation between TAG content and lipid unsaturation degree at the population level as measured by LC-MS. Each dot represents one culture under the N- conditions (each triplicate at 6, 12, 24, 48, 72 and 96 hours). Click here for file
