# Supplementary figures and images for: Quantitative dynamics of triacylglycerol accumulation in microalgae populations at single-cell resolution revealed by Raman microspectroscopy
Source: Biotechnol Biofuels. 2014 Apr 9;7:58. doi: 10.1186/1754-6834-7-58 (PMC4022372; doi:10.1186/1754-6834-7-58)

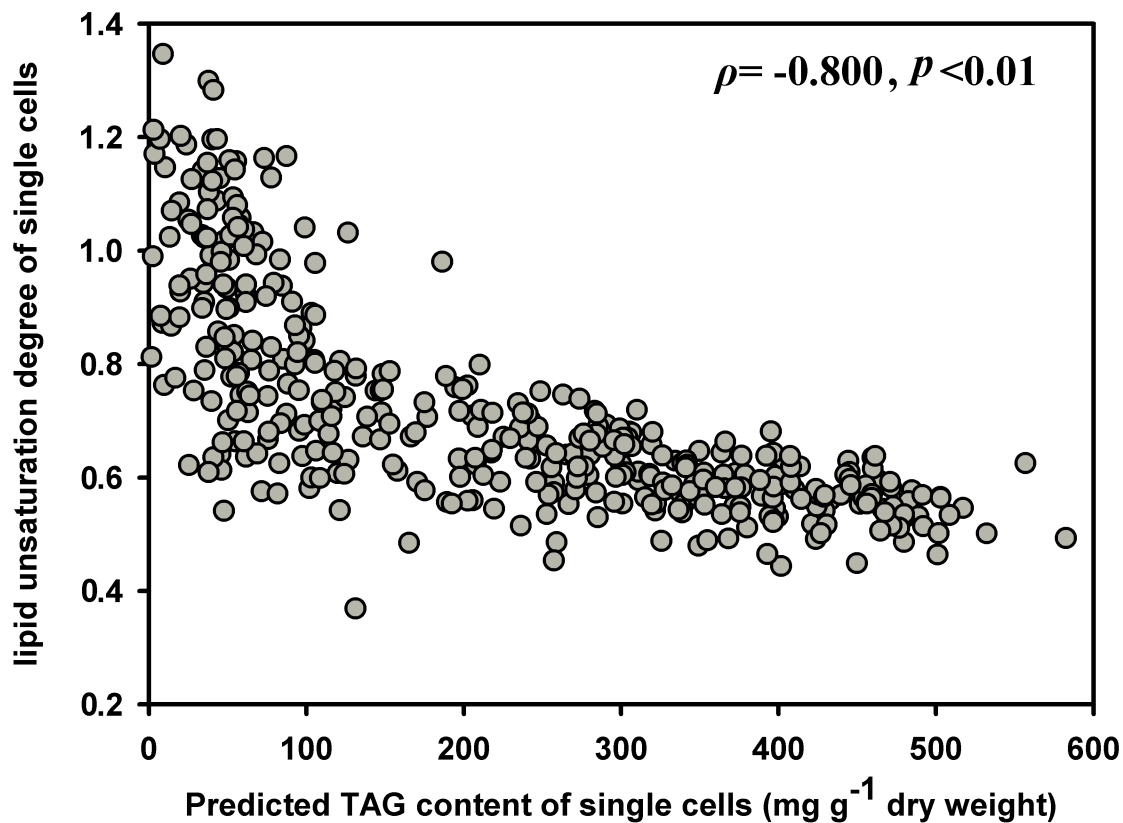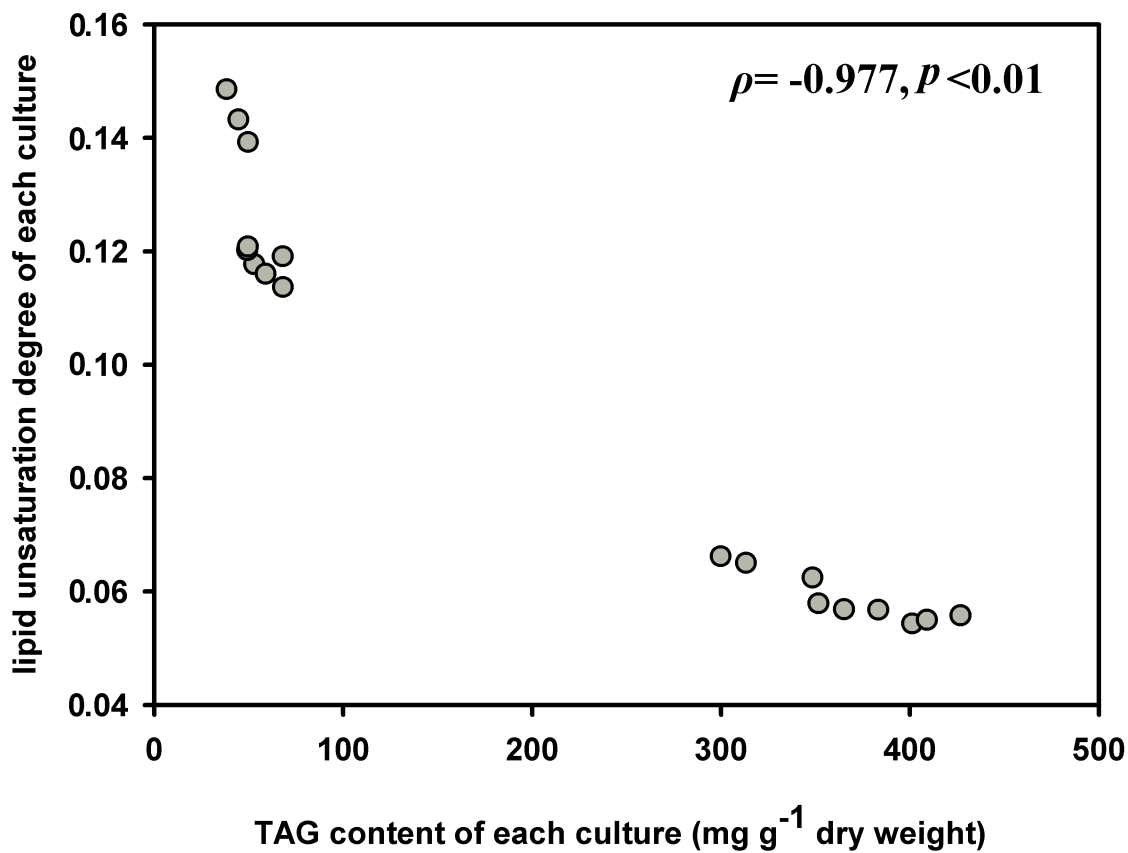

Supplement: Additional file 3 — Correlation between the TAG content and lipid unsaturation degree at single-cell level and population level. (A) Correlation between TAG content and lipid unsaturation degree at the single-cell level as measured by SCRS. Each dot represents one cell in the N- cultures. (B) Correlation between TAG content and lipid unsaturation degree at the population level as measured by LC-MS. Each dot represents one culture under the N- conditions (each triplicate at 6, 12, 24, 48, 72 and 96 hours). [file 1754-6834-7-58-S3.pdf]
